# Obsessive passion, opportunity recognition, and entrepreneurial performance: The dual moderating effect of the fear of failure

**DOI:** 10.3389/fpsyg.2022.1037250

**Published:** 2023-01-04

**Authors:** Yuqi Tu, Xiling Hao, Joanna Rosak-Szyrocka, László Vasa, Xin Zhao

**Affiliations:** ^1^School of Economics and Management, Yanshan University, Qinhuangdao, China; ^2^School of Business Administration, Anhui University of Finance and Economics, Bengbu, China; ^3^Department of Production Engineering and Safety, Faculty of Management Czestochowa University of Technology, Częstochowa, Poland; ^4^School of Economics, Széchenyi István University, Győr, Hungary; ^5^School of Statistics and Applied Mathematics, Anhui University of Finance and Economics, Bengbu, China

**Keywords:** obsessive passion, opportunity recognition, fear of failure, entrepreneurial performance, entrepreneurs

## Abstract

A strong inclination toward an important or preferred activity is a critical factor that drives individual to engage in corresponding activities. This study focuses on how entrepreneurs, influenced by obsessive passion, are motivated to put great effort into taking advantage of business opportunities and accomplishing entrepreneurial goals. By using SPSS and AMOS tools to analyze the multi-source questionnaire of 208 entrepreneurs, the research results show that obsessive passion can promote entrepreneurial performance and opportunity recognition plays a mediating role. In addition, endogenous and exogenous fear of failure play different moderating roles in the effect of obsessive passion on opportunity recognition. The research conclusion deepens the theoretical understanding of entrepreneurial passion, opportunity recognition, and fear of failure at a more subtle level.

## 1. Introduction

Passion is crucial for entrepreneurial progress (Cardon et al., 2009; Li et al., 2020). Despite pervasive pressure, passionate entrepreneurs can mobilize their creativity and continue to establish their businesses. Some become addicted to it and work tirelessly around the clock. Entrepreneurs who are labeled “workaholics,” “addicts,” etc. are often influenced by a strong inclination to act on preferences or important issues. As an uncontrollable passion to engage in an activity (Vallerand et al., 2003; Cardon et al., 2013; Fu et al., 2022), the strong behavioral inclination of obsessive passion is of practical importance in stimulating the desire to explore, motivate involvement, mobilize entrepreneurial behavior (Vasa, 2002; Murad et al., 2021), and even affect entrepreneurial consequences.

The nature of obsessive passion is that it is repetitive, uncontrollable, and based on long-term behavioral inclination and preference (Vallerand et al., 2003; Mageau et al., 2005; Thorgren and Wincent, 2015; Fu et al., 2022). Obsessive-passionate individuals will put considerable time and effort into a job or task in order to gain social respect, maintain good external relations, and achieve performance requirements (Vallerand et al., 2003, 2007; Karácsony et al., 2020; Stroe et al., 2020). Some studies have pointed out that most entrepreneurs are driven by obsessive passion to perform entrepreneurial actions (Thorgren and Wincent, 2015).

Although previous studies have demonstrated that passion can stimulate individuals to action in order to achieve anticipated goals (Murnieks et al., 2016; Fu et al., 2022), there is still limited theoretical comprehension and empirical evidence to demonstrate how obsessive passion can motivate entrepreneurs’ behaviors and affect their results. Firstly, we know little about how obsessive passion drives individual behavior, especially in the entrepreneurial context (Stroe et al., 2020). Entrepreneurship is the process of developing business opportunities to achieve a return on entrepreneurial performance. It is necessary to explore the mechanism of the influence of obsessive passion on the recognition of entrepreneurial opportunity as well as entrepreneurial performance. Second, our understanding is limited as to whether obsessive passion has a positive impact in the entrepreneurial process (Fu et al., 2022). Theoretical research indicates that obsessive passion is often associated with negative outcomes in daily life (Siren et al., 2016; Stroe et al., 2020; Bayraktar and Jiménez, 2022). However, obsessive passion plays an important role in focusing on work attention, increasing job involvement, and stimulating exploration desires (Thorgren and Wincent, 2015). This study builds a model of “obsessive passion-opportunity recognition-entrepreneurial performance” to understand how obsessive passion influences the response of entrepreneurs and the developmental effects of entrepreneurial ventures.

Moreover, obsessive passion attaches great importance to the controlled internalization process. This is when individuals internalize their activities into their individual identity under the influence of external pressure (Vallerand et al., 2003; Cardon et al., 2013). Entrepreneurs with obsessive passion not only require complete accomplishment of entrepreneurial goals to achieve and support their own values, but also need to meet the attention and expectations of significant stakeholders, such as key partners, venture capitalists, and even family members (Vallerand et al., 2003, 2007). Therefore, the fear of failure in entrepreneurial actions is inevitable (Bélanger et al., 2013). Based on this, to further consider the influence of fear of failure on the identification and exploration of opportunities for entrepreneurs with obsessive passion, this study introduces fear of failure as the boundary condition of the influence of obsessive passion on opportunity recognition and considers its moderating role.

The structure of this article is as follows. First, a literature review is conducted to describe the research in respect of obsessive passion, opportunity recognition, fear of failure, and entrepreneurial performance. Second, we propose the research hypothesis. We then describe the research design and the specific empirical analysis process of this study. Finally, the theoretical model of obsessive passion, opportunity, and entrepreneurial performance and future perspectives are discussed. Specifically: (1) This study explores the effect of obsessive passion on entrepreneurial performance. It attempts to open the “black box” of the mechanism of obsessive passion and to deepen the theory of entrepreneurial passion and entrepreneurial performance. (2) We analyze the mediating effect of opportunity recognition in the relationship between obsessive passion and entrepreneurial performance. This is done in order to deepen the understanding of the process of entrepreneurial opportunity recognition, and to also enrich the previous research results of entrepreneurial cognition and behavior. (3) This paper explains the moderating effects of endogenous and exogenous fear of failure on obsessive passion and opportunity recognition. This will help explain the various action pathways of different types of fear of failure from a more subtle perspective.

## 2. Literature review

### 2.1. Obsessive passion

The dualistic theoretical model of passion (Vallerand et al., 2003; Cardon et al., 2013; Bayraktar and Jiménez, 2022) defines passion as a strong inclination toward a self-defining activity that one loves, finds important, and invests a significant amount of time and energy in. Compared to harmonious passion, obsessive passion refers to a controlled internalization of an activity into one’s identity (Vallerand et al., 2003). The pivotal distinction between obsessive passion and harmonious passion is whether the urge to engage in the activity remains under the individual’s control (Fu et al., 2022). Individuals with obsessive passion generally have an obvious inclination to engage in target activities and behaviors, frequently feel “cannot help but to engage in the passionate activity” (Vallerand et al., 2003; Patel et al., 2015; Fu et al., 2022).

This reveals the controlled internalization process of an individual’s external motivation to engage in an activity. This internalization comes from a variety of pressures, both from something attached to the activity—such as obtaining social recognition or fulfilling performance requirements—and because individuals feel that the excitement brought about by participating in the activity becomes uncontrollable (Vallerand et al., 2003; Klaukien et al., 2013). In turn, external pressures control the individual and compel him or her to pursue the activity to achieve and sustain these outcomes (Stroe et al., 2020). Therefore, the higher the obsessive passion for an activity, the more an individual cannot resist the internal impulse to participate in the activity. This may also cause the individual to attach great importance to the target activity and pay ample time, energy, and resources (Lalande et al., 2017; Warnick et al., 2018; Fu et al., 2022), as well as stimulate the desire to explore, constantly consider, and explore the novelty of the entrepreneurial process; and stimulate the degree of engagement, which is specifically manifested as long-term and continuous entrepreneurial behavior.

### 2.2. Opportunity recognition

Opportunity recognition is a core prerequisite for entrepreneurship and business development. It is a complex process (Shane and Venkataraman, 2000; Shepherd et al., 2022) that reflects the comprehensive ability of entrepreneurs and start-ups to perceive the key components of entrepreneurship, such as the target market, user demand, resource acquisition, and environmental dynamics. Entrepreneurs and entrepreneurial organizations identify potentially feasible opportunities from various ideas, and continuously develop these opportunities. In this process, the potential value of opportunities is constantly reviewed, and entrepreneurs’ strategic positioning of opportunities is clarified. Indeed, opportunity recognition is not simply a cognitive process (McMullen and Shepherd, 2006; Gregoire et al., 2010). It also embodies a behavioral response process that includes judgment, focus, and evaluation.

### 2.3. Fear of failure

The fear of failure is a psychological construct to explain the interaction between entrepreneurs’ cognitive, emotional, and motivational attributes in the context of achievement; and is complicated (Cacciotti et al., 2016, 2020). Entrepreneurial activities are affected by obstacles, setbacks, and difficulties, which bring the risk of potential failure. It is inevitable that they will provoke an emotional experience and cognitive response to the fear of failure (Kollmann et al., 2017; Stroe et al., 2020). This fear of entrepreneurial failure is individual reactive and context-dependent. It is a consequence of internal and external interactions. Not only will individuals worry about the possible negative effects of failure on the self out of an internal ego need, but they will also worry about the negative evaluations of important others, namely endogenous and exogenous fear of failure (Hao et al., 2022).

Endogenous fear of failure is focused on the entrepreneur’s internal self. It is fear that a self-centered evaluation of failure may bring adverse consequences to oneself. When encountering the obstacles of entrepreneurial frustration, entrepreneurs induce endogenous fear of failure from the perspective of internal attribution of self-worth and ability. It is associated with feelings of shame and embarrassment, and fear of devaluing self-worth. The latter takes account of external interpersonal relationships, that is, focusing on others and emphasizing the fear triggered by changes in the connection between oneself and the surrounding relationships. Business activities are based on external resources, relationship networks, and business communication (Wennberg et al., 2013). In the process of continuous contact with the outside world in a social network, entrepreneurs are prone to worry and be anxious about not being valued or even being unable to positively influence stakeholders and other important people. Due to fear of failure, individuals spare no effort to avoid adverse effects on their own behavior and subsequently affect the expected results and goals (Cacciotti and Hayton, 2015; Kollmann et al., 2017).

### 2.4. Entrepreneurial performance

As a consequence of the enterprise fulfilling certain tasks or attaining certain goals in the process of entrepreneurship (Sandberg and Hofer, 1987), entrepreneurial performance will reflect the success or failure of entrepreneurial activity. Entrepreneurial performance is a scale and criterion for judging and testing the results of business activities. It is usually used to describe the achievements of entrepreneurial enterprises and the potential for future market growth (Bates, 1998). Specifically, it is not only related to the ability of the firm to achieve break-even rate of operation, but also reflects the competitive advantage of the firm and indicates the possibility of further development (Chrisman et al., 1998).

In conclusion, through a series of activities centered around entrepreneurship, enterprises will achieve diverse results that reflect their start-up and growth. This study understands entrepreneurial performance as a key indicator of whether an enterprise can further survive and grow. It focuses on obsessive passion as the critical factor in the study of the relationship between obsessive passion-opportunity recorecognition and entrepreneurial performance” to obsessive passion-opportunity recognition-entrepreneurial performance. It is beneficial to explore the internal mechanism that drives entrepreneurial performance.

## 3. Hypothesis development and research model

### 3.1. The influence of obsessive passion on entrepreneurial performance

Obsessive passion means that individuals have a strong inclination to work and participate in activities which make them unable to control themselves. They therefore continuously commit to work and activities (Vallerand et al., 2003; Fu et al., 2022). In the process of constantly seeking business success, entrepreneurs with obsessive passion will improve their level of entrepreneurial effort, promote entrepreneurial persistence, and thus facilitate entrepreneurial performance.

At the cognitive level, entrepreneurs with obsessive passion are stimulated by various pressures to devote considerable time and energy (Vallerand et al., 2007) in order to achieve high performance in their entrepreneurial activities. They will devote attention and process information related to the survival and development of the enterprise, concentrating their attention and cognition on entrepreneurial performance. Vallerand et al. (2003) demonstrated that individuals with obsessive passion fully focus on the task at hand, have an intensely strong inclination to achieve the goal, and are not distracted by other things. This focus on entrepreneurial activities can effectively achieve and promote entrepreneurial performance.

At the motivation level, obsessive-passionate entrepreneurs often focus on performance, interpersonal relationships, and social expectations (Vallerand et al., 2007). When individual identity and self-concept are integrated, the entrepreneur is prone to carry out activities consistent with their identity, such as entrepreneurs and leaders. They derive excitement from them, constantly stimulated by the power and ideas to engage in entrepreneurial behaviors. They usually continue to commit to entrepreneurship with a strong passion to achieve entrepreneurial performance goals. In conclusion, entrepreneurs with obsessive passion can boost their entrepreneurial performance by recognizing their focus and motivation to achieve their goals. Thus, we propose the following hypothesis:

*H1*: Obsessive passion has a positive effect on entrepreneurial performance.

### 3.2. The relationship between obsessive passion and opportunity recognition

The recognition of entrepreneurial opportunities is usually a reflection of the entrepreneur’s psychology and other subtle elements (Gregoire et al., 2010). When entrepreneurs seek opportunities to achieve their entrepreneurial goals and external expectations, they can mobilize their cognition, alertness, depth, and breadth of information collection. They will use this to promote the possibility of identifying potential opportunities.

At the cognitive level, entrepreneurs with high obsessive passion are likely to devote considerable time and energy to entrepreneurial activities (Stroe et al., 2020). Entrepreneurship is crucial to them, as they tend to seek opportunities to prove themselves. This cognitive belief provides a major impetus for continuous exploration of opportunities.

At the motivation level, individuals with a strong inclination to complete certain tasks are usually characterized by evaluation orientation, repetitive thinking, and a keen awareness of market changes. In this way, key opportunity clues can be captured, and potential business opportunities can be found more easily than others. They will use their contacts and connections, evaluation skills, and judgment (Gaglio and Katz, 2001; Ardichvili et al., 2003). In summary, entrepreneurs with obsessive passion are more capable of recognizing opportunities than are those without obsessive passion. Based on the above analysis, we proposed the following hypothesis:

*H2*: Obsessive passion has a positive effect on opportunity recognition.

### 3.3. The mediating role of opportunity recognition

The creative use of opportunities to create value is a key to promoting performance and achieving entrepreneurial success (Shane and Venkataraman, 2000). The process of entrepreneurs searching for valuable information in the external environment and identifying opportunities is an important source of potential business opportunities. This also lays the foundation for subsequent value creation. Only by finding suitable business opportunities can enterprises survive and achieve good performance (Donbesuur et al., 2020).

From a cognitive perspective, opportunity recognition is the process by which entrepreneurs perceive and conceptualize opportunities in a complex business environment. They do this to realize value increments (Gregoire et al., 2010), stimulate the generation of new wealth, and thus promote entrepreneurial performance.

In addition, from the perspective of motivation, the objective of the opportunity recognition behavior of entrepreneurs is to achieve both entrepreneurial success and enterprise survival and development. To realize innovation, sustainability, and feasibility, the active and alert exploration of these potential opportunities can help enterprises establish competitive advantages in the market. It will help them avoid being imitated by others, and obtain higher returns on performance (Donbesuur et al., 2020). In summary, opportunity recognition often leads entrepreneurs to find profitable and feasible business opportunities that play a crucial role in entrepreneurial performance, and there is a positive correlation between them. Based on the reasoning above, we propose the following:

*H3*: Opportunity recognition has a positive effect on entrepreneurial performance.

Opportunity recognition is an important behavioral manifestation of obsessive passion and is also a vital driver of entrepreneurial performance. Obsessive passion can motivate entrepreneurs to put sufficient effort into capturing opportunity-related information and recognizing opportunities. This will lead them to explore and achieve entrepreneurial performance goals through the identified opportunities.

At the cognitive level, obsessive-passionate entrepreneurs who commit considerable time and energy to their businesses tend to be more sensitive to the outside world. They pay more attention to various types of information in terms of cognition and attention (Vallerand et al., 2007), and gather a wider range of information. Comprehensive, large-scale, and heterogeneous information significantly increases the potential for opportunity recognition. It also provides more valuable opportunities, and makes subsequent opportunity exploitation more effective, thereby promoting entrepreneurial performance.

At the level of motivation, entrepreneurs with obsessive passion care about their external views and social expectations (Vallerand et al., 2007). Interpersonal pressure, social expectations, and external attention catalyze the attachment of importance to social networks, which are key sources of entrepreneurial resources and information acquisition (Birley, 1985). Individuals with such resources are better able to effectively recognize opportunities, effectively promote enterprises that are profitable and promote entrepreneurial performance (Donbesuur et al., 2020). In conclusion, the strong inclination of obsessive passion is reflected in the response to opportunity recognition. As a key linkage, opportunity recognition will eventually affect entrepreneurial performance. Thus, this study proposes the following hypothesis:

*H4*: Opportunity recognition mediates the relationship between obsessive passion and entrepreneurial performance.

### 3.4. The moderating role of fear of failure

Endogenous fear of failure reflects embarrassment and shame, and internal fear of devaluing self-worth. From a cognitive perspective, this inner-negative perception stimulates entrepreneurs to pay more attention to internal adjustments and moderation. However, these individuals have limited cognitive and psychological resources (Donbesuur et al., 2020). Focusing limited cognitive resources on internal regulation will reduce attention to external interpersonal relationships and social recognition. It will further weaken the intense behavioral inclination to constantly seek and explore opportunities that focus on social respect and recognition from others.

From the perspective of motivation, the motivation for opportunity recognition driven by obsessive passion mainly comes from the external pressure to obtain social recognition or fulfill performance requirements. The inclination of individuals to feel that the excitement brought about by participating in activities then becomes uncontrollable (Klaukien et al., 2013). The inherent fear induces negative emotional experiences and the shame and embarrassment of entrepreneurs (Mcgregor and Elliot, 2005; Stroe et al., 2020). This further weakens motivation and excitement, hindering their focus on the task at hand. It also prevents them from engaging in full emotional and effective processing of external information searching and opportunity exploration. Based on the above reasoning, we contend that under the influence of limited cognitive resources and negative affections, endogenous fear of failure weakens the positive relationship between obsessive passion and opportunity recognition. Consequently, this study proposes the following hypothesis:

*H5a*: Endogenous fear of failure negatively moderates the relationship between obsessive passion and opportunity recognition.

The exogenous fear of failure, which starts with external relations and takes others as the core, emphasizes an individual’s external recognition of others, social status, and other concerns (Hao et al., 2022). The process by which obsessive passion stimulates the entrepreneur to turn external pressure into motivation is influenced by this exogenous fear of failure. It encourages the recognition of the possibilities of opportunity from a cognitive perspective, damaged social status, and negative social evaluation of cognition will stimulate the entrepreneur to draw on their cognitive resources. They will also apply time and effort to tackle the pressure and use it to safeguard the interests of the external network, protect their social status, and constantly look for new opportunities and potential business opportunities (Hao et al., 2022). It enhances the obsessive passion of the individual’s uncontrollable action preference caused by external pressure, and then promotes the possibility of opportunity recognition.

From the perspective of motivation, focusing on external factors, such as social identity, stimulates the motivation of individuals to deal with external relevance events (Bernabé et al., 2014). The obsessive passion reflected by this motivational inclination (Vallerand et al., 2003) can stimulate the search for external information, and valuable clues. It further improves the belief in finding opportunities that may achieve external expectations, and also enhances the positive effect of obsessive passion on opportunity recognition. Above all, the exogenous fear of failure, which focuses on relationships and connections with others, has a positive moderating effect on the positive relationship between obsessive passion and opportunity recognition. The higher the fear of exogenous failure, the more entrepreneurs will produce behavioral preferences that focus on social recognition and respect. Therefore, this study proposes the following hypothesis:

*H5b*: Exogenous fear of failure positively moderates the relationship between obsessive passion and opportunity recognition.

**Figure 1:**
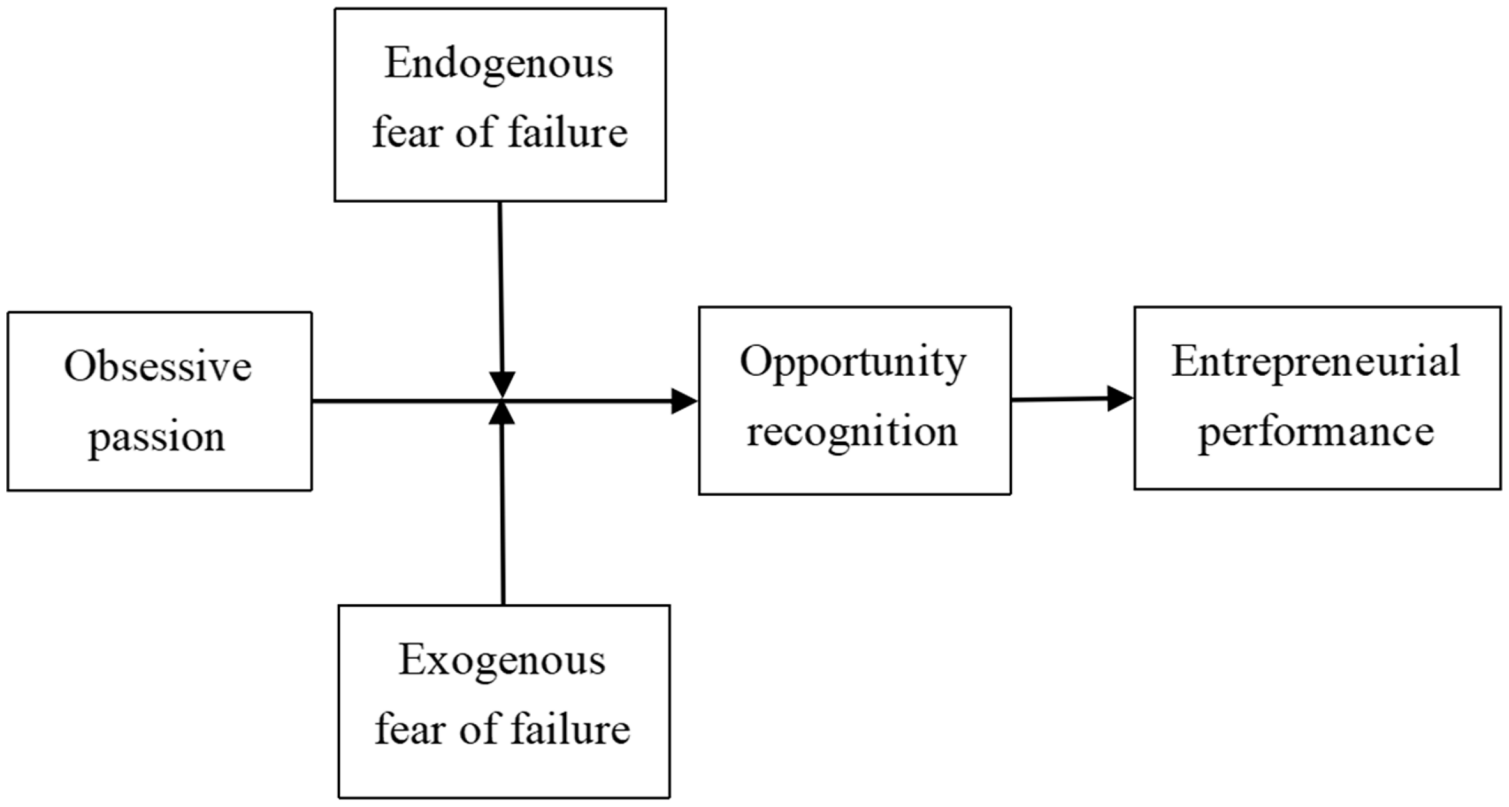
Research model.

Based on the aforementioned analysis, this study built a theoretical model of obsessive passion, opportunity recognition, fear of failure, and entrepreneurial performance, as shown in Figure 1.

## 4. Materials and methods

### 4.1. Sample and procedures

Following Amezcua et al. (2013), this study defines entrepreneurial ventures as those established for no more than 5 years. The main participants in the research were the founders of startups. We used a variety of techniques for data collection, including: (1) investigated the start-up parks and incubators in Jiangsu, China. We conducted field inspections with the help of Chinese entrepreneurs’ exchange meetings, and distributed questionnaires with the help of the principals and staff; (2) used professional research institutions to conduct questionnaire distribution and data collection; and (3) mobilized various contacts to contact entrepreneurs, and invited the respondents to fill in the questionnaire by means of QR code scanning and link transmission.

The entire data collection process focused on the details of completing the time, manner, place, questionnaire number, and other information. Contact was maintained with research institutions and enterprises throughout the process, tracking the progress of the survey, and reviewing the feedback. The entire sample data collection process took half a year. The survey subjects covered Beijing, Shanghai, Jiangsu, and other Chinese regions with a high entrepreneurial activity index. 638 questionnaires were collected. After careful screening, 208 valid questionnaires were accepted, yielding an effective questionnaire recovery rate of 32.6%.

The samples were normally distributed and were an even representation. The proportions of male and female entrepreneurs were 67.3 and 32.7%, respectively. This is roughly consistent with the proportion of male and female entrepreneurs in the GEM database. In terms of age and education, 70.7% of the respondents had a bachelor’s degree or above, and most (72.6%) of the respondents were under 35 years old. This indicating that most entrepreneurs in this survey were young people with higher education. Additionally, more than 70% of the respondents said they were married, and 88.9% of the respondents (88.9%) started small and minor businesses.

### 4.2. Measures

#### 4.2.1. Obsessive passion

Murnieks et al. (2014) used the obsessive passion part of the binary passion scale developed by Vallerand et al. (2003), which has good reliability and validity. There are seven measurement items; the example items are “The urge is so strong. I cannot help myself from doing this entrepreneurial activity” and “I have difficulty imagining my life without this entrepreneurial activity.” Scores were scored using a five-point Likert scale, from 1 to 5 representing a range from “strongly disagree” to “strongly agree.”

#### 4.2.2. Opportunity recognition

To measure opportunity recognition, we referred to the scale of Gregoire et al. (2010), including “the degree of alignment between an opportunity means of supply and target markets” and “the perceptions of an opportunity general feasibility.” Specifically, we used five items, such as “The proposed business solution can be used to solve the problems of the targeted market” and “Applying the proposed business solution with individuals/firms in the targeted market constitutes a feasible opportunity.” Respondents were asked to choose the option that best reflected the real situation based on the relevant situation of the start-up, ranging from 1 = “not at all” to 5 = “a great deal.”

#### 4.2.3. Fear of failure

Fear of failure was measured based on Performance Failure Appraisal Inventory (PFAI) of Conroy et al. (2002), which is one of the most widely used scales of fear of failure. Many scholars have used it to explore individual psychological and behavioral activities (Wood and Pearson, 2009; Mitchell and Shepherd, 2011; Leon and Anna, 2018). To capture the forms and dimensions of fear of failure in an entrepreneurial context through field research and data analysis, a scale of entrepreneurial fear of failure was developed and verified in entrepreneurial practice. It measured a total of 16 items to capture two potential common factors (Hao et al., 2022). Among them, the endogenous fear of failure included “When I am failing, I am afraid that I might not have enough talent” and other five items. And a representative item for exogenous fear of failure is “When I am not succeeding, my value decreases for some people.” The measurements used five-point rating scales anchored 1 = “does not correspond at all to me” to 5 = “corresponds exactly to me.”

#### 4.2.4. Entrepreneurial performance

Referring to the measurement methods used by Liu et al. (2019), the performance measurements included annual profit, sales, market share, and growth rate of new employees. It specifically included “The annual growth rate of enterprise sales compared with the local average level of the same industry” and three other metrics. The use of these relative performance indicators yielded more accurate and objective results.

#### 4.2.5. Control variables

It generally includes individual and enterprise-level variables, such as the entrepreneur’s gender, age, education, marital status, number of start-ups, pre-entrepreneurial work experience and time, as well as enterprise size, industry, and region (Ozgen and Baron, 2007; Khan et al., 2017, 2019a; Liu et al., 2019; Stroe et al., 2020).

## 5. Results

### 5.1. Reliability and validity

All the variables were tested for reliability and validity (Table 1). The results of the reliability analysis showed that Cronbach’s α coefficient for each variable was above 0.8. This indicates that the questionnaire had a high reliability. The factor loadings, composite reliabilities (CRs), and average variance extracted (AVE) values were higher than the recommended coefficient weights (Fornell and Larcker, 1981).

**Table 1:** Validity and reliability tests of the main variables.

Variable	Questionnaire item	Factor loading	AVE	CR	Cronbach’s α
Obsessive passion	I cannot live without it.	0.729	0.584	0.906	0.876
	The urge is so strong. I cannot help myself from doing this entrepreneurial activity.	0.785			
	I have difficulty imagining my life without this entrepreneurial activity.	0.767			
	I am emotionally dependent on this entrepreneurial activity.	0.544			
	I have a tough time controlling my need to do this entrepreneurial activity.	0.859			
	I have almost an obsessive feeling for this entrepreneurial activity.	0.834			
	My mood depends on me being able to do this entrepreneurial activity.	0.789			
Opportunity recognition	The proposed business solution can be used to solve the problems of the targeted market.	0.806	0.603	0.884	0.836
	The proposed business solution has the capabilities to answer the needs of the market described.	0.820			
	There is a “match” between what the proposed business solution does, and what the targeted market demands.	0.756			
	Applying the proposed business solution with individuals/firms in the targeted market does constitute a feasible opportunity	0.723			
	The proposed business solution is sufficiently developed to be applied with individuals/firms in the targeted markets.	0.775			
Entrepreneurial performance	The annual growth of new employees compared with the local average level of the same industry.	0.805	0.747	0.922	0.887
	The annual growth rate of enterprise sales compared with the local average level of the same industry.	0.882			
	The annual growth in market shares compared with the local average level of the same industry.	0.910			
	The annual growth rate of profits compared with the local average level of the same industry.	0.857			
Endogenous fear of failure	When I am not succeeding, I am less valuable than when I succeed.	0.693	0.514	0.863	0.810
	When I am not succeeding, I get down on myself easily.	0.742			
	When I am failing, I feel embarrassed and ashamed of myself.	0.702			
	When I am failing, it is often because I am not smart enough to perform successfully.	0.656			
	When I am failing, I blame my lack of talent.	0.766			
	When I am failing, I am afraid that I might not have enough talent.	0.735			
Exogenous fear of failure	When I am not succeeding, people are less interested in me.	0.640	0.569	0.929	0.915
	When I am not succeeding, people seem to want to help me less.	0.759			
	When I am not succeeding, people tend to leave me alone.	0.774			
	When I am not succeeding, some people are not interested in me anymore.	0.781			
	When I am not succeeding, my value decreases for some people.	0.788			
	When I am failing, it upsets important others.	0.748			
	When I am failing, important others criticize me.	0.731			
	When I am failing, I lose the trust of people who are important to me.	0.778			
	When I am failing, important others are not happy.	0.730			
	When I am failing, important others are disappointed.	0.803			

Confirmatory factor analysis was conducted using AMOS 24.0. Table 2 shows that the fitting degree of the five-factor model F5 is optimal, and all fitting indices are within a reasonable range, indicating that the questionnaire has relatively ideal external construct validity, and the follow-up analysis can be continued.

**Table 2:** Results of confirmatory factor analysis.

Model	Factor	χ^2^	Df	χ^2^/df	RMSEA	CFI	TLI	IFI
One-factor model F1	O + E1 + E2 + R + P	2554.443	464	5.505	0.148	0.393	0.351	0.399
Two-factor model F2a	O + E1 + E2 + R, P	2085.782	463	4.505	0.130	0.529	0.495	0.533
Two-factor model F2b	O + E1 + E2, R + P	1944.048	463	4.199	0.124	0.570	0.539	0.574
Three-factor model F3	O, E1 + E2, R + P	1278.255	461	2.773	0.093	0.763	0.745	0.765
Four-factor model F4	O, E1 + E2, R, P	669.230	458	1.461	0.047	0.536	0.497	0.572
Five-factor model F5	O, E1, E2, R, P	626.596	437	1.434	0.046	0.945	0.938	0.946

*N* = 208; O, R, E1, E2, and P represent obsessive passion, opportunity recognition, endogenous fear of failure, exogenous fear of failure, and entrepreneurial performance, respectively.

### 5.2. Descriptive statistics

The mean, standard deviation, and correlation coefficient of each variable are listed in Table 3. The results showed that the correlation coefficients between obsessive passion, opportunity, and entrepreneurial performance are 0.314 and 0.192, respectively, indicating a positive correlation between obsessive passion and opportunity recognition and entrepreneurial performance. The correlation coefficient between opportunity recognition and entrepreneurial performance is 0.411 (*p* < 0.01), indicating a positive correlation between opportunity recognition and entrepreneurial performance.

**Table 3:** Correlation and descriptive statistics.

	1	2	3	4	5	6	7	8	9	10	11	12	13	14	15
1	1														
2	−0.179^**^	1													
3	−0.198^**^	0.241^***^	1												
4	−0.070	0.515^***^	0.065	1											
5	−0.161^**^	0.237^**^	−0.041	0.162^**^	1										
6	−0.091	0.237^**^	0.094	0.106	0.186^**^	1									
7	−0.102	0.635^***^	0.043	0.401^***^	−0.020	0.018	1								
8	0.116	−0.063	0.037	−0.019	0.016	0.090	−0.133^*^	1							
9	−0.005	−0.255^***^	−0.109	−0.208^**^	−0.156^**^	−0.109	−0.094	0.097	1						
10	−0.007	−0.057	0.002	0.015	0.061	0.004	0.016	0.048	0.040	1					
11	−0.022	0.028	0.018	0.009	0.129^*^	0.001	−0.059	−0.025	−0.159^**^	−0.019	1				
12	−0.087	0.134^*^	0.236^**^	−0.038	0.072	0.300^***^	0.023	0.017	−0.103	0.037	0.314^***^	1			
13	0.098	−0.217^**^	−0.302^***^	−0.091	−0.114	−0.123^*^	−0.131^*^	0.045	0.151^**^	0.104	−0.213^**^	−0.136^*^	1		
14	−0.128^*^	0.047	−0.083	0.096	0.168^**^	−0.084	0.048	0.049	0.051	0.088	0.217^**^	−0.024	0.055	1	
15	0.076	0.072	0.038	0.119^*^	0.167^**^	0.142^**^	−0.020	−0.079	−0.234^**^	−0.062	0.292^***^	0.411^***^	−0.074	−0.027	1
M	1.330	33.330	2.820	2.260	1.510	2.160	1.710	9.320	4.460	2.870	3.528	3.884	3.151	2.925	2.929
SD	0.470	5.829	0.824	0.880	0.742	0.427	0.737	0.964	0.748	0.792	0.691	0.608	0.699	0.751	0.892

^*^p < 0.1, ^**^p < 0.05, ^***^p < 0.01; 1 = gender, 2 = age, 3 = education, 4 = marriage status, 5 = entrepreneurial times, 6 = pre-entrepreneurial work experience, 7 = pre-entrepreneurial working time, 8 = industry types, 9 = enterprise scale, 10 = regional position, 11 = obsessive passion, 12 = opportunity recognition, 13 = endogenous fear of failure, 14 = exogenous fear of failure, and 15 = entrepreneurial performance.

### 5.3. Hypothesis testing

#### 5.3.1. Mediating effect analysis

This study used two approaches to test the mediating role of opportunity identification (Fritz and MacKinnon, 2007; MacKinnon et al., 2007). Using test steps of Baron and Kenny (1986) as a reference, the cross-level regression method was used to verify the research hypothesis, and the hypothesis test results of each model are shown in Table 4. Models 1 and 5 describe the relationships between the control variables, entrepreneurial performance, and opportunity recognition. Model 2 confirms a significant positive correlation between obsessive passion and entrepreneurial performance (β = 0.256, *p* < 0.01), thus supporting Hypothesis 1. Model 6 shows that obsessive passion has a significantly positive effect on opportunity recognition (β = 0.301, *p* < 0.01), supporting Hypothesis 2. Model 3 shows a positive correlation between opportunity recognition and entrepreneurial performance (β = 0.440, *p* < 0.01), thus verifying Hypothesis 3. The results of Model 4 show that when the independent variable (obsessive passion) and the mediating variable (opportunity recognition) are simultaneously included in the regression model at the same time, opportunity recognition still has a significant positive impact on entrepreneurial performance (β = 0.392, *p* < 0.01). However, the positive effect of obsessive passion on entrepreneurial performance is weakened (β = 0.138, *p* < 0.05). Therefore, opportunity recognition partially mediates the relationship between obsessive passion and entrepreneurial performance. Next, based on Preacher and Hayes (2004), this study uses the bootstrapping method to further examine the mediating effect of opportunity recognition between obsessive passion and entrepreneurial performance. The results of the analysis show that the indirect effect of opportunity recognition on entrepreneurial performance was 0.1131 (with a confidence interval: 0.0501 to 0.1955 at 95% CI and does not cross the zero value) in 5,000 number of bootstrap samples. This finding indicates that opportunity recognition has a significant mediating effect on the relationship between obsessive passion and entrepreneurial performance (*p* < 0.01). Therefore, Hypothesis 4 is supported.

**Table 4:** Hypothesis test results.

Variable	Entrepreneurial performance				Opportunity recognition			
	Model 1	Model 2	Model 3	Model 4	Model 5	Model 6	Model 7	Model 8
Gender	0.312^*^	0.304^*^	0.322^**^	0.316^**^	−0.022	−0.031	−0.076	−0.020
Age	−0.009	−0.011	−0.014	−0.014	0.010	0.008	0.014	0.006
Education	0.075	0.071	−0.034	−0.024	0.248^**^	0.244^**^	0.237^**^	0.247^**^
Marriage status	0.116	0.127	0.187^**^	0.186^**^	−0.162^*^	−0.149^**^	−0.159^*^	−0.154^*^
Entrepreneurial times	0.207^*^	0.168	0.192^**^	0.172^*^	0.034	−0.012	−0.028	0.001
Pre-entrepreneurial work experience	0.356^*^	0.387^**^	0.072	0.119	0.647^**^	0.683^***^	0.738^***^	0.680^***^
Pre-entrepreneurial working time	−0.064	−0.034	−0.076	−0.059	0.028	0.063	0.048	0.103
Industry types	−0.037	−0.033	−0.034	−0.032	−0.008	−0.003	−0.003	0.001
Enterprise scale	−0.258^**^	−0.208^**^	−0.222^**^	−0.199^**^	−0.081	−0.022	0.011	−0.011
Regional position	−0.069	−0.067	−0.094	−0.091	0.058	0.060	0.061	0.075
Obsessive passion		0.256^***^		0.138^**^		0.301^***^	0.306^***^	0.353^***^
Opportunity recognition			0.440^***^	0.392^***^				
Endogenous fear of failure							0.068	
Endogenous fear of failure^*^Obsessive passion							−0.136^**^	
Exogenous fear of failure								−0.064
Exogenous fear of failure^*^Obsessive passion								0.107^*^
*R* ^2^	0.120	0.180	0.274	0.290	0.142	0.231	0.248	0.249
Adj.*R*^2^	0.074	0.132	0.232	0.245	0.097	0.187	0.196	0.197
*F* stat	2.596^**^	3.786^***^	6.525^***^	6.429^***^	3.164^***^	5.191^***^	4.774^***^	4.799^***^

^*^p < 0.1, ^**^p < 0.05, and ^***^p < 0.01.

#### 5.3.2. Moderating effect analysis

**Figure 2:**
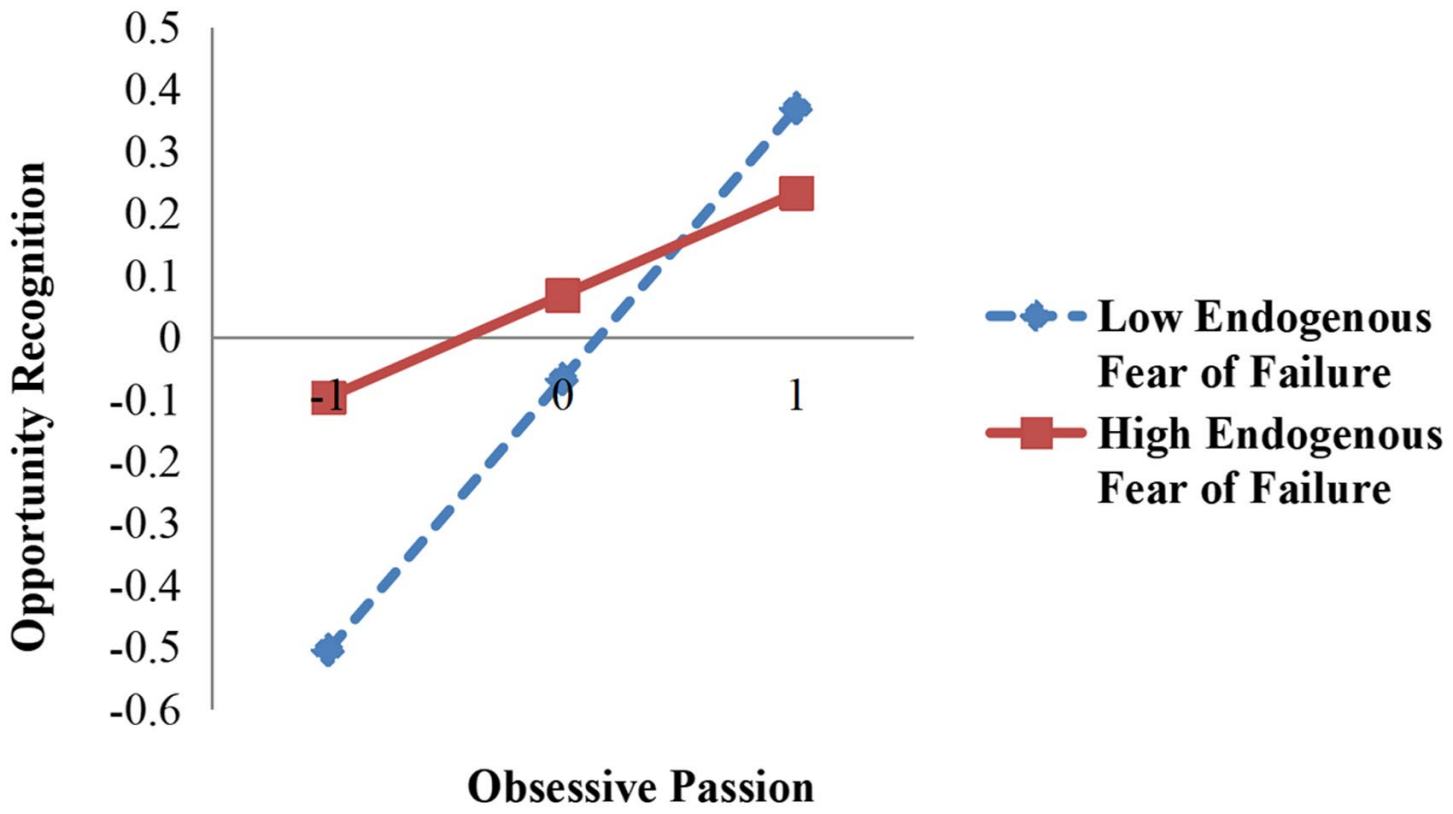
Moderating effect of endogenous fear of failure between obsessive passion and entrepreneurial performance.

**Figure 3:**
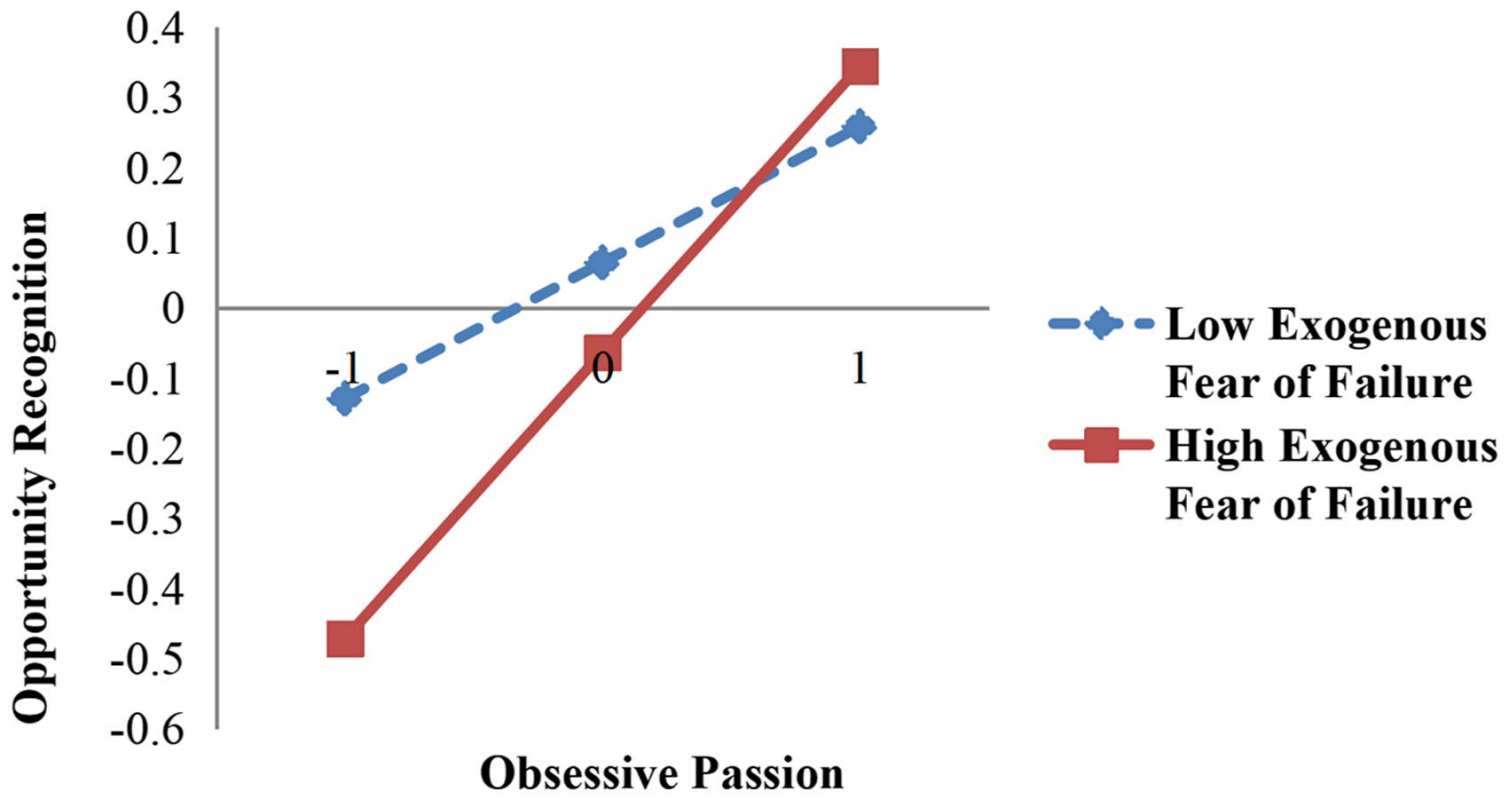
Moderating effect of exogenous fear of failure between obsessive passion and entrepreneurial performance.

To test the moderating effect, hierarchical regression was conducted by constructing the interaction terms. Models 7 and 8 show the moderating effect of the fear of failure. Endogenous fear of failure had a negative moderating effect on the relationship between obsessive passion and opportunity recognition (β = −0.136, *p* < 0.05). Thus, Hypothesis 5A is supported. Exogenous fear of failure had a positive moderating effect on the relationship between obsessive passion and opportunity recognition (β = 0.107, *p* < 0.1), thus verifying Hypothesis 5B. The moderating effects are shown in Figures 2, 3.

### 5.4. Robustness tests

To further clarify the robustness of the test results, we combined the following two auxiliary means for testing: On the one hand, obsessive passion means that individuals will feel stressed and invest considerable energy and emotion urgently and strongly in related activities in order to achieve the set goals. Therefore, this kind of “urgency” and “intensity” are particularly significant in the behavior and activities of obsessive-passionate entrepreneurs. This study therefore selected “unable to hold oneself back” and “obsession,” as the two factors that can most interfere with individual actions. The two variables were reanalyzed using regression as a new variable. On the other hand, because the underlying goal of venture enterprises is to earn profits and survive, survival is the absolute index for measuring entrepreneurial performance (Chrisman et al., 1998). Therefore, we select “annual profit, sales and market share,” which are more representative of “profitability,” to verify whether the research hypothesis is consistent with expectations. Using the changed independent variable “obsessive passion,” and dependent variable “entrepreneurial performance” measurement items for detection and analysis, it was found that the original main effect of “obsessive passion-opportunity recognition-entrepreneurial performance” and the two-sided moderating effects of endogenous and exogenous fear of failure are still significant, as shown in Table 5 below, verifying the stability of the conclusions of this study.

**Table 5:** Robustness tests.

Variable	Entrepreneurial performance				Opportunity recognition			
	Model 1	Model 2	Model 3	Model 4	Model 5	Model 6	Model 7	Model 8
Gender	0.233	0.217	0.237^*^	0.229	−0.010	−0.031	−0.071	−0.020
Age	−0.012	−0.011	−0.016	−0.016	0.011	0.011	0.016	0.010
Education	0.118	0.109	−0.033	−0.024	0.353^**^	0.341^**^	0.340^**^	0.348^**^
Marriage status	0.090	0.092	0.163^*^	0.158^*^	−0.172^*^	−0.170^*^	−0.175^*^	−0.176^*^
Entrepreneurial times	0.214^**^	0.174^*^	0.200^**^	0.181^**^	0.032	−0.019	−0.034	−0.009
Pre-entrepreneurial work experience	0.296	0.292	0.025	0.046	0.635^**^	0.631^***^	0.664^***^	0.622^**^
Pre-entrepreneurial working time	−0.072	−0.061	−0.082	−0.076	0.024	0.037	0.020	0.061
Industry types	−0.037	−0.033	−0.034	−0.032	−0.006	−0.002	−0.001	0.000
Enterprise scale	−0.214^**^	−0.175^*^	−0.179^**^	−0.161^*^	−0.083	−0.034	−0.009	−0.020
Regional position	−0.070	−0.074	−0.095	−0.095	0.058	0.054	0.060	0.066
Obsessive passion		0.231^**^		0.118^*^		0.291^***^	0.300^***^	0.327^***^
Opportunity recognition			0.426^***^	0.390^***^				
Endogenous fear of failure							0.057	
Endogenous fear of failure^*^Obsessive passion							−0.130^*^	
Endogenous fear of failure								−0.058
Endogenous fear of failure^*^Obsessive passion								0.096^*^
*R* ^2^	0.106	0.156	0.268	0.280	0.152	0.229	0.243	0.243
Adj.*R*^2^	0.059	0.108	0.225	0.234	0.108	0.184	0.190	0.190
*F* stat	2.258^**^	3.201^**^	6.319^***^	6.118^***^	3.433^***^	5.118^***^	4.634^***^	4.630^***^

^*^p < 0.1, ^**^p < 0.05, and ^***^p < 0.01.

## 6. Discussion

Obsessive passion has a significant impact on entrepreneurial behavior and results (Thorgren and Wincent, 2015). Previous studies have shown that individuals with obsessive passion usually have greater “obsession” with work and tasks and tend to invest significant time and energy (Vallerand et al., 2007) in order to complete goals, achieve external expectations, and cope with pressure. However, it is not clear how obsessive passion works, especially in entrepreneurial contexts. This is because entrepreneurs turn extrinsic stress into intrinsic motivation and drive themselves to achieve entrepreneurial excellence. This study takes opportunity recognition as a mediating variable, and tests the relationship between obsessive passion and entrepreneurial performance. It then introduces endogenous and exogenous fear of failure to explore the boundary effects of obsessive passion and opportunity recognition. Through an empirical test of large-scale data, the study finds that the inclination of obsessive passion positively affects entrepreneurial performance, and opportunity recognition is a key link between obsessive passion and entrepreneurial performance. Fear of failure is a “double-edged” sword in the mechanism of obsessive passion and opportunity recognition. Endogenous fear of failure weakens the positive effect of obsessive passion on opportunity recognition, while exogenous fear of failure, which focuses on the relationship and connection with others, enhances the positive relationship between obsessive passion and opportunity recognition.

### 6.1. Theoretical contributions

Starting from obsessive passion to explore its influence, this study not only expands the understanding of the effect of obsessive passion, but also enriches the theoretical scope of entrepreneurial passion. At present, most related research on entrepreneurial passion explores it as an overall concept, and a few empirical studies attaching importance to a specific passion are mostly focused on the level of harmonious passion. The results of this study further broaden the theoretical application scope of obsessive passion and contribute to a more comprehensive understanding of entrepreneurial passion.

Moreover, in the passion literature, obsessive passion—that is, the strong inclination of an individual to engage in an activity—has been typified by labels such as “work-life imbalance,” “authoritarian decision-making,” or “physical and mental health problems” (Vallerand et al., 2003; Mol et al., 2019). The few previous studies of obsessive passion tended to view it as the “dark side” of passion (Siren et al., 2016; Stroe et al., 2020), concentrating on the negative effects of obsessive passion on individuals and rarely paying attention to its positive effects on entrepreneurs. In fact, obsessive passion is frequently accompanied by entrepreneurs who have a certain incentive and stimulation effect. For example, Thorgren and Wincent (2015) state that more entrepreneurs are driven by obsessive passion to conduct entrepreneurial activities continuously. This study finds that the positive effect of obsessive passion plays a key role in stimulating entrepreneurs to take initiative, which can effectively stimulate entrepreneurs to recognize opportunities and promote entrepreneurial performance. Therefore, this study provides new insights on the content of obsessive passion. It deeply explores the positive impact of obsessive passion on opportunity recognition and entrepreneurial performance. It also enriches the existing research results on the “positive side” of obsessive passion.

Opportunity recognition is introduced as a mediating variable to analyze the path mechanism of the impact of obsessive passion on entrepreneurial performance. This further deepens the understanding of the mechanism between the two and strengthens the understanding of the process of opportunity recognition by entrepreneurs and their causes and consequences. Previous studies on the individual influencing factors of opportunity recognition mainly emphasize the input resources, such as time, money, and effort of entrepreneurs (Mitchell and Shepherd, 2011). Entrepreneurs’ individual characteristics, such as prior experience, creativity, and cognitive capacity (Shepherd and Detienne, 2005; Tumasjan and Braun, 2011), were also reviewed. From the perspective of obsessive passion, this study considers the relationship between the obsessive passion and the process of entrepreneurial opportunity recognition. It enriches the exploration of entrepreneurial psychology regarding the mechanism of opportunity recognition. In addition, the conclusion of the positive relationship between opportunity recognition and entrepreneurial performance further shows that entrepreneurial opportunities need to be profitable (Shane and Venkataraman, 2000). Opportunity recognition lays the foundation for the subsequent development of opportunities to promote entrepreneurial performance. By identifying potentially effective opportunities, entrepreneurs can obtain sustainable profits and achieve corporate survival.

Research on the double-sided effect of fear of failure clarifies the boundary conditions of the relationship between obsessive passion and opportunity recognition. This adds a new explanation for appreciating their internal logic. It also shows that fear of failure are a “double-edged sword,” which can offer a more comprehensive understanding of the construct and influence of fear of failure. According to Cacciotti et al. (2016), fear of failure is multifaceted, and its behavioral reactions are not limited to negative aspects, such as inhibition and depression. It can also have motivative and incentive effects. This study also proves that endogenous and exogenous fear of failure play diverse roles in the relationship between obsessive passion and opportunity recognition. The former weakens the positive relationship between obsessive passion and opportunity recognition, whereas the latter enhances the positive effect of obsessive passivity on opportunity recognition. In contrast with previous research findings on the subject, which showed that fear of failure is not conducive to the identification and utilization of opportunities, hindering entrepreneurial behavior, and suppressing entrepreneurial performance (Kollmann et al., 2017; Stroe et al., 2020), the results of this study provide a new logic and empirical verification to explain how fear of failure exerts different influential effects.

### 6.2. Managerial implications

First, entrepreneurs need to understand the psychological status of obsessive passion and turn external pressure into internal motivation. They then need to improve their entrepreneurial effort under the stimulation of moderate obsessive passion in order to boost their subsequent opportunity recognition and entrepreneurial performance. The psychology of entrepreneurs has an important impact on entrepreneurial behavior (Shepherd, 2004). This study breaks away from previous cognition and identifies the advantages of obsessive passion, which plays a key role in stimulating opportunities to identify and achieve entrepreneurial achievements. Therefore, entrepreneurs need to face their own obsessive passion, adjust their own state, and reasonably turn pressure into motivation under moderate obsessive passion. Second, we corrected the bias of fear of failure and examined its impact of fear of failure in a targeted manner. The fear of failure from different sources has different ways and means of action. Therefore, it is vital to bravely face realistic challenges and inner fears, analyze the content of different levels of fear of failure, and exert the motivational effect of fear of failure. Third, entrepreneurship education trainers and workers should pay attention to the psychological conditions of entrepreneurs, such as obsessive passion and fear of failure. It is essential to attach importance to internal psychological guidance, and fully mobilize the initiative of entrepreneurs. Simultaneously, it is important to correct their cognitive prejudices, see a dialectical view of the influence of obsessive passion and fear of failure, and provide help when entrepreneurs seek opportunities and pursue goals.

### 6.3. Limitations and future directions

This study has three main limitations that need to be further explored and improved in the future: (1) The impact of obsessive passion and fear of failure may vary according to entrepreneurs’ social, economic, and cultural environments (Molina-Ramírez and Barba-Sánchez, 2021; Barba-Sánchez et al., 2022). Therefore, cross-border comparisons may help analyze the impact of national and policy environments on enterprise behavior and performance (Khan et al., 2019b), as well as the boundary role of fear of failure. (2) Cross-sectional data was used for this empirical analysis. In future, the impact of time should be considered. Dynamic data on passion, fear of failure, opportunity recognition, and entrepreneurial performance changes can be obtained through cross-stage and multi-point tracking—in order to more comprehensively explore the dynamic process of passion. (3) This study mainly used questionnaires to obtain first-hand data and adopted a self-report measurement method to collect data. Although various measures have been taken to reduce deviation and data deviation tests have been conducted, it is difficult to overcome the inherent defects of questionnaires and retrospective studies. In future research, we can consider the comprehensive use of experiments, case studies, and other methods for more accurate analysis.

## 7. Conclusion

Obsessive passion plays an important role in the competitive business environment and is the key factor driving entrepreneurs to engage in entrepreneurial activities. By tracking how entrepreneurs can facilitate opportunity recognition and promote entrepreneurial performance under the influence of obsessive passion, this study expands current research on entrepreneurial psychology, cognition, and behavior. Our discoveries that endogenous and exogenous fear of failure play dual moderating roles in the effect of obsessive passion on opportunity recognition provide a way to understand the effect of fear of failure at a more subtle level. We hope that this study will spur more research on obsessive passion, opportunity recognition and fear of failure.

## Data availability statement

The original contributions presented in this study are included in the article/supplementary material, and further inquiries can be directed to the corresponding author.

## Ethics statement

Ethical review and approval were not required for the study of human participants, in accordance with local legislation and institutional requirements. Written informed consent for participation was not required for this study, in accordance with national legislation and institutional requirements. The patients/participants provided online informed consent to participate in this study, which stated the voluntary nature of participation and assurance of confidentiality and anonymity.

## Author contributions

YT: conceptualization, methodology, data curation, analysis, and original draft preparation. XH: writing—validation and formal analysis. JR-S: writing—review and editing. LV: supervision. XZ: investigation and funding acquisition. All authors contributed to the article and approved the submitted version.

## Funding

This research was supported by the Graduate Innovation Funding Project of Hebei Province (Grant No.CXZZBS2023045), the National Natural Science Foundation of China (Grant No.72072001), the Innovation Development Research Project of Anhui Province (Grant No.2021CX053), and the Teaching Research Project of Anhui University of Finance and Economics (Grant No. acszjyyb2021111).

## Conflict of interest

The authors declare that the research was conducted in the absence of any commercial or financial relationships that could be construed as potential conflicts of interest.

## Publisher’s note

All claims expressed in this article are solely those of the authors and do not necessarily represent those of their affiliated organizations, or those of the publisher, the editors and the reviewers. Any product that may be evaluated in this article, or claim that may be made by its manufacturer, is not guaranteed or endorsed by the publisher.
